# Electrochemical DNA biosensors developed for the monitoring of biointeractions with drugs: a review

**DOI:** 10.55730/1300-0527.3584

**Published:** 2023-09-30

**Authors:** Arzum ERDEM, Huseyin ŞENTÜRK, Esma YILDIZ, Meltem MARAL, Ayla YILDIRIM, Aysen BOZOĞLU, Burak KIVRAK, Neslihan Ceren AY

**Affiliations:** Analytical Chemistry Department, Faculty of Pharmacy, Ege University, İzmir, Turkiye

**Keywords:** Electrochemical biosensor, DNA biosensor, drug-DNA interaction, intercalation, anticancer drug

## Abstract

The interaction of drugs with DNA is important for the discovery of novel drug molecules and for understanding the therapeutic effects of drugs as well as the monitoring of side effects. For this reason, many studies have been carried out to investigate the interactions of drugs with nucleic acids. In recent years, a large number of studies have been performed to electrochemically detect drug–DNA interactions. The fast, sensitive, and accurate results of electrochemical techniques have resulted in a leading role for their implementation in this field. By means of electrochemical techniques, it is possible not only to demonstrate drug–DNA interactions but also to quantitatively analyze drugs.

In this context, electrochemical biosensors for drug–DNA interactions have been examined under different headings including anticancer, antiviral, antibiotic, and central nervous system drugs as well as DNA-targeted drugs.

An overview of the studies related to electrochemical DNA biosensors developed for the detection of drug–DNA interactions that were reported in the last two decades in the literature is presented herein along with their applications and they are discussed together with their future perspectives.

## 1. Introduction

Biosensors are small devices that can detect the target analyte specifically, allowing selective analysis. They have advantages such as small size, low cost, high selectivity, wide measurement range, low detection limit, fast response, practical use, and high stability. Biosensors are widely used in the healthcare field for the detection of biological molecules, pathogens, and other disease-causing agents. The transducer converts the information from the interaction between the biolayer and analyte into a measurable signal, while the biolayer is responsible for the specific interaction [1].

A diverse array of transducers can be utilized to transform the interplay between an analyte and a bioreceptor into an observable signal. These transducers work based on different principles, with mass sensitive transducers converting the interaction into a change in bending or resonance frequency, optical transducers detecting changes in light frequency or intensity, and electrochemical transducers generating a signal based on the change in potential. The choice of transducer depends on several factors, including the type of analyte and bioreceptor under consideration, as well as the specific requirements of the detection system. Such considerations are crucial when developing accurate and effective biosensors, which have a wide range of applications in different fields, including biomedical research, environmental monitoring, and food safety. In the final stage, the reading system measures the amounts of these changes [2].

A bioreceptor is a biological entity that can be either a molecular species or a living biological system and that utilizes a biochemical mechanism to identify a specific substance or analyte. Among the diverse interactions employed, antigen–antibody interactions, nucleic acid interactions, enzymatic interactions, cellular interactions, and biomimetic or synthetic bioreceptors are the most frequent types employed. Bioreceptors, a molecule or molecular structure that has a specific binding affinity or interaction with another molecule, are protein- or nucleic acid-based. Bioreceptors such as enzymes, nucleic acids, antibodies, and cells serve as vital components in various fields, including medical diagnostics, environmental monitoring, and food safety, among others [3,4].

Biosensors are commonly divided into subgroups according to the type of conversion system used: piezoelectric biosensors, thermal biosensors, optical biosensors, and electrochemical biosensors. The components of electrochemical sensors are the reference electrode, working electrode, and auxiliary electrode, separated by an electrolyte [5].

Electrochemical biosensors are simple electronic devices capable of taking rapid measurements. They have the ability to monitor environmental conditions and determine analyte concentrations at a given point in a complex sample. Electrochemical biosensors have several advantages that make them attractive diagnostic devices in medicine [6].

For biosensing applications, electrochemical techniques like potentiometry, impedimetry, and amperometry can be utilized through different types of sensors [7]. Electrochemical impedance spectroscopy (EIS) is a valuable technique that provides insights into the physicochemical alterations that occur on the surface of an electrode when analytes bind to a bioreceptor immobilized on the electrode. Alternatively, a potentiometric biosensor is capable of detecting potential changes that occur during the formation of interactions between bioreceptors and analytes [8]. When applying voltammetry technique as an electrochemical analytical method, the current is observed in relation to changes in electrode potential. Moreover, amperometry involves measuring the current over time while maintaining a consistent potential, making it a method that quantifies the concentration of electroactive species undergoing oxidation or reduction at the working electrode. In coulometry-based studies, the primary signal converter can be a glassy carbon electrode (GCE), Au electrode, indium tin oxide electrode, pencil graphite electrode (PGE), and screen-printed carbon electrode (SPCE).

An electrochemical biosensor is a molecular sensing device that closely connects a biological recognition element to an electrode transducer. The purpose of the transducer is to convert the biological recognition event into a readable electrical signal [1].

Direct electrochemical detection of DNA interactions is essential for the development of facile, rapid, and user-friendly DNA detection devices [9,10].

**Figure 1:**
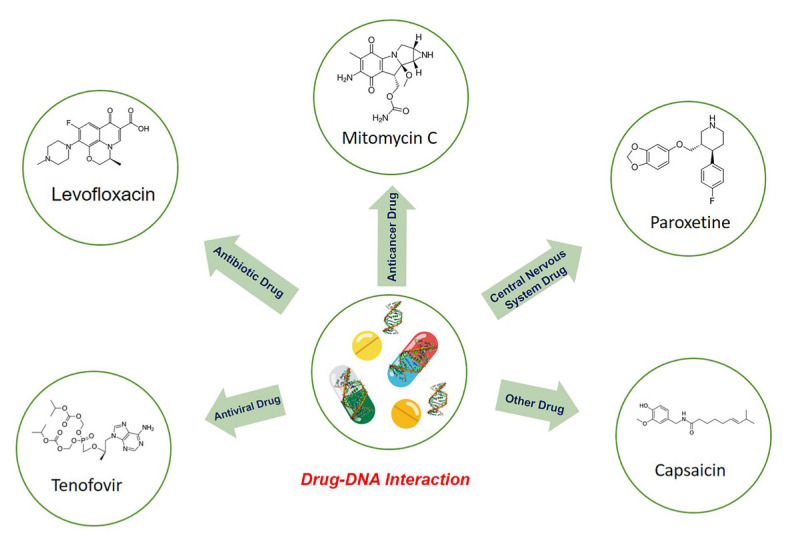
Interactions of different types of drug with DNA.

With the discovery of the electroactivity of DNA [11], many studies have been carried out for the electrochemical determination of nucleic acids. This initiated the electrochemical determination of drug–DNA interactions and made it possible to electrochemically investigate the interactions of many different types of drugs with DNA (Figure 1) [10,12–27]. There are recent reports on electrochemical studies using various nanomaterials based on the mechanism of drug–DNA interactions in the literature [28–30].

Drugs can interact with DNA in several ways, including intercalation, groove binding, DNA cleavage, covalent binding, nucleoside analog incorporation, cross-linking, and noncovalent interactions [31–39]. The drug molecules may target the edges of the bases of DNA as well as its sugar-phosphate backbone, or the double helix structure of DNA [40–42]. Structural changes in the double helix DNA occur after drug interaction, leading to thermodynamic stability and functional changes. Drug–DNA interactions occur in mainly three different ways: (i) drug molecules connect to DNA through transcription factors and polymerases, (ii) RNA binds to the DNA double helical structure, forming a nucleic acid triple helical structure, and (iii) drug molecules connect to the DNA double helical structure through different mechanisms such as connecting to the negatively charged nucleic sugar-phosphate structure, intercalating between the bases, or interacting with the minor and major grooves of the DNA [10].

## 2. Electrochemical detection of drug–DNA interactions

The interaction between a drug and DNA is studied by many different techniques such as chromatographic, spectroscopic, optic, and electrochemical. Traditional methods have some disadvantages such as being time-consuming, the low sensitivity of some of them, the expensive equipment required, and the need for trained personnel. The most important advantages of electrochemical techniques are that they are fast and sensitive, suitable for miniaturization, easy to use, and allow on-site analysis. In addition, due to the presence of electroactive groups in some drug molecules, drug analysis and interactions with DNA can be easily demonstrated specifically. For these reasons, the number of studies focused on this field has been increasing day by day. In this section, electrochemical biosensors for drug–DNA interactions are classified and examined according to drug types. In this context, the studies on the development of biosensors for monitoring the interactions of anticancer, antiviral, antibiotic, and central nervous system drugs with DNA are overviewed. The advantages, disadvantages, difficulties, and applications of these studies are discussed.

### 2.1 The interactions of anticancer drugs/candidate drugs with DNA

Cancer is the one of the main causes of death today. Today, there are many different processes for the treatment of cancer. The cure rate increases with early diagnosis of cancer and initiation of drug therapy. Primary induction therapy for advanced disease or cancers without more effective treatment approaches; surgery or radiotherapy for those with localized disease; and surgery, radiotherapy, and local treatment methods covering both of these are applied. Today, there are many drugs according to the type of cancer and different treatment methods are still being investigated. Anticancer drugs usually target DNA and kill cancerous cells. Therefore, the interaction of the anticancer drug with DNA is very important. Further, examining the interaction of the drug with DNA is of great importance for the discovery of new drug molecules [43]. In this section, electrochemical biosensor studies screening the interactions between anticancer drugs and DNA are presented.

#### 2.1.1 Nucleic acid biosensors for monitoring the interaction of drugs with nucleic acids

Nucleic acid biosensors are powerful tools for monitoring the interaction of drugs with nucleic acids. These biosensors are designed to detect changes in the structure, conformation, or activity of nucleic acids in response to drug binding [1,9,10,12].

The electrochemical biosensor can measure the changes in the electrochemical properties of the nucleic acid probe in response to drug binding. Multiple electrochemical methods, including cyclic voltammetry (CV), differential pulse voltammetry (DPV), and electrochemical impedance spectroscopy (EIS) can be utilized to achieve this goal. Nucleic acid biosensors show substantial promise for drug discovery and development applications due to their ability to furnish crucial insights into the binding affinity, specificity, and mechanism of action of drugs that target nucleic acids.

**Figure 2:**
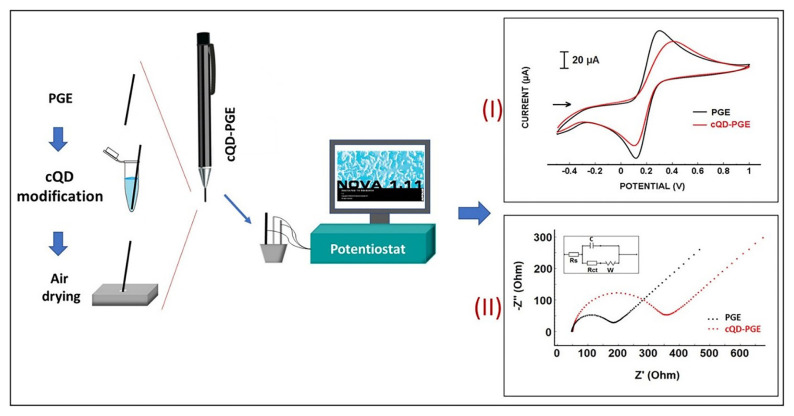
The concept of fabrication of the cQD modified pencil graphite electrodes. Left, the sketch of the cQD modification onto the surface of PGE. cQD modification is performed by passive adsorption by dipping PGEs into the cQD solution during 1 h. Then cQD-PGEs were air dried for 10 min before electrochemical measurements. (I) Cyclic voltammograms of PGE and cQD-PGE. Supporting electrolyte solution is 0.1 M KCl containing 2 mM [Fe(CN)_6_]^3−/4−^. (II) Nyquist diagrams of PGE and cQD-PGE. Supporting electrolyte solution is 0.1 M KCl containing 2.5 mM [Fe(CN)_6_]^3−/4−^. Inset is the equivalent circuit model used for fitting of the impedance data. Reprinted from Ref. [31], Copyright (2020), with permission from Elsevier.

The interaction of DNA with the anticancer drug daunorubicin was determined by using DPV in combination with a PGE modified with carbon quantum dots [31]. The studies on the characterization of a PGE modified with carbon quantum dots developed for the DNA–drug interaction were carried out using CV and EIS (Figure 2). The drug–DNA interaction was analyzed based on the changes in the guanine oxidation signal measured voltammetrically.

Javar et al. developed an electrochemical DNA biosensor for the detection of the interaction of the anticancer drug amsacrine with DNA. The carbon paste electrode (CPE) surface was firstly modified with Eu^3+^ doped NiO and amsacrine detection was performed via DPV [44]. Under optimized conditions, the limit of detection (LOD) was calculated as 0.05 μM in the range of 0.1–100 μM.

In a recent study by Nimal et al., an electrochemical biosensor was developed for monitoring the DNA interaction with the antileukemia drug azacytidine [32]. A GCE was used in the study and the drug–DNA interaction was investigated through the change in guanine and adenine signals measured by DPV.

Abedi et al. developed an electrochemical biosensor for the interaction between epirubicin and prostate cancer-related nucleic acid sequence [45]. In the study, graphite screen-printed electrodes were used and they were modified with gold nanocubes. The interaction of epirubicin with DNA at different concentrations was investigated by DPV. In addition, the application of the developed biosensor in human blood serum samples was demonstrated.

Karimi-Maleh et al. developed a DNA biosensor for monitoring the interaction of DNA with the anticancer drug idarubicin [33]. Their investigation involved the modification of a GCE by Pt- and Pd-incorporated ZnO nanoparticle-decorated single-wall carbon nanotube nanocomposites, followed by the immobilization of DNA onto the modified electrode surface. Upon interaction of the DNA with idarubicin, the changes in the guanine oxidation signal were detected using DPV. The LOD was calculated as 0.8 nM within the range of 1.0 nM–65 μM. Additionally, molecular docking studies were performed, which validated the interaction of DNA with the drug.

Findik et al. synthesized hybrid nanoflowers with glycine and L-lysine amino acids [34]. Then the characterization of the hybrid nanoflowers was performed with FTIR, Raman spectroscopy, scanning electron microscopy (SEM), energy-dispersive X-ray spectroscopy (EDX), and X-ray photoelectron spectroscopy (XPS). The PGE surface was modified with synthesized nanoflowers and then calf thymus double-stranded DNA was immobilized onto the nanoflower-modified electrode surface. The interaction of the anticancer drug mitomycin C with DNA was examined using a glycine nanoflower-modified electrode via DPV.

**Figure 3:**
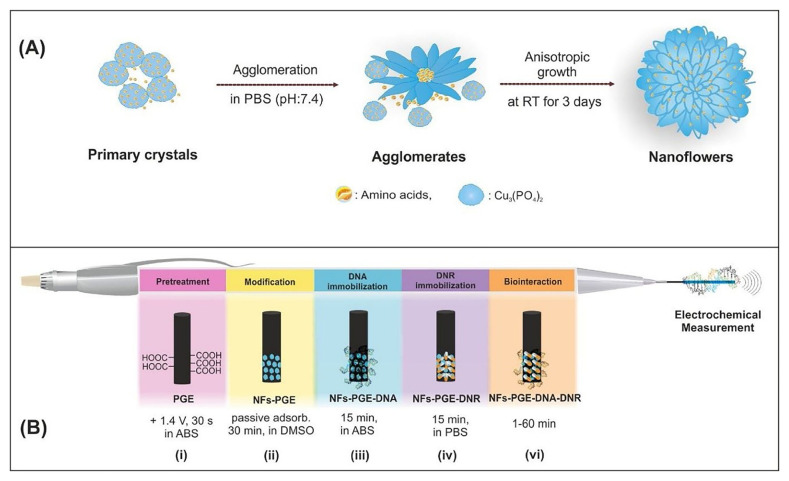
(A) Schematic illustration of the formation of amino acids-Cu_3_(PO_4_)_2_ hybrid NFs, (B) The representative scheme of the pretreatment of PGE (i), modification of NFs (ii), immobilization of DNA (iii) and DNR (iv), surface-confined interaction of DNR and ctdsDNA (vi). Abbreviations: DNR: daunorubicin, RT: room temperature. Reprinted from Ref. [46], Copyright (2021), with permission from Elsevier.

Findik et al. developed modified pencil graphite electrodes (NFs-PGEs) composed of organic/inorganic hybrid nanoflowers to perform electrochemical analysis of the interaction between the anticancer drug daunorubicin and calf thymus double-stranded DNA (ctdsDNA) as shown in Figure 3 [46]. In their study, electrochemical measurements were carried out using DPV and EIS and, accordingly, the LOD for daunorubicin (DNR) was calculated as 2.93 μM and 2.06 μM for L-cysteine nanoflowers-modified PGE (c-NFs-PGE) and L-glutamic acid nanoflowers-modified PGE (ga-NFs-PGE), respectively.

Another electrochemical biosensor was presented for the investigation of the anticancer drug mitomycin C–DNA interaction [47]. In that study, a multiwalled carbon nanotube-modified PGE was used and microscopic and electrochemical characterization studies were performed with SEM and CV. Before and after the interaction of drug and DNA, the guanine oxidation signal was measured and the drug–DNA interaction was investigated.

In another study, the interaction of mitomycin C and ctdsDNA was investigated by using a chitosan/ionic liquid composite-modified PGE [48]. In that study, mitomycin C and DNA oxidations signals were measured in the same potential range.

An electrochemical sensor for the determination of another anticancer drug, mitoxantrone, was introduced by Saljooqi et al. [49]. The GCE surface was modified with multiwalled carbon nanotube silver nanoparticles and polythiophene nanocomposite and DNA was immobilized onto the modified electrode surface and, accordingly, mitoxantrone was detected via DPV. In addition, mitoxantrone was determined in urine and blood serum samples.

Machini et al. developed an electrochemical DNA biosensor for the investigation of the interaction of nivolumab with DNA [50]. The drug–DNA interaction was investigated with several techniques such as DPV, EIS, gel electrophoresis, UV-Vis spectrophotometry, and quartz crystal microbalance.

**Figure 4:**
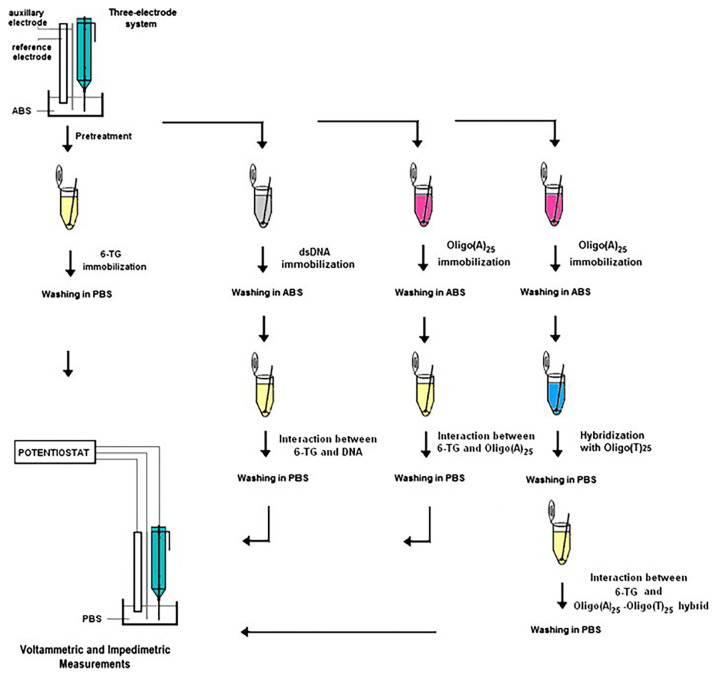
Experimental presentation for the step-by-step modification of PGEs with 6-TG, DNA, Oligo (A)_25_ or the double helix of Oligo (A)_25_–Oligo (T)_25_ and voltammetric and impedimetric detection of interaction between 6-TG and DNA (or 6-TG and Oligo (A)_25_, or 6-TG and Oligo (A)_25_–Oligo (T)_25_ double helix) at the PGE surface. Reprinted from Ref. [51], Copyright (2014), with permission from Elsevier.

A single-use PGE was used for detecting the interaction between 6-thioguanine (6-TG) and double-stranded DNA (dsDNA) through the use of two electrochemical techniques (Figure 4): DPV and EIS [51]. The results of impedimetric analysis performed for the interaction between 6-TG and dsDNA were consistent with the voltammetric results. The authors also performed DPV in order to measure the oxidation signals produced by 6-TG and adenine in oligonucleotides as (A)25–Oligo (T)25. Previous studies have explored the use of various types of electrodes for detecting interactions between drugs and DNA, but their study showed that a PGE-based sensor could provide sensitive and cost-effective monitoring of the interaction between 6-TG and DNA without the need for surface modifications using nanomaterials such as CNTs.

**Figure 5:**
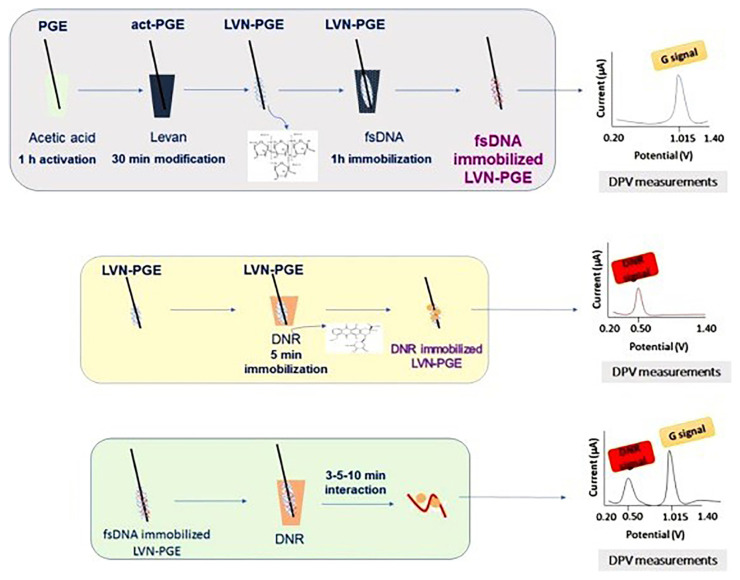
The experimental steps of the modification of LVN at PGE surface, voltammetric determination of fsDNA and DNR using LVN-PGE and the voltammetric analysis of the biomolecular interaction between fsDNA and DNR at LVN-PGE surface. Reprinted from Ref. [35], Copyright (2021), with permission from Elsevier.

Daunorubicin (DNR), an antineoplastic agent widely employed in cancer therapy, exhibits antitumor activity through intercalation between the base pairs of DNA upon interaction with DNA. Levan-modified PGEs (LVN-PGEs) were developed for the first time and used for the investigation of the interaction between DNR and DNA [35]. Fish sperm DNA was immobilized on the surface of LVN-PGE, and the interaction between DNA and DNR was investigated using DPV (Figure 5). It was reported that the LOD for the detection of DNR on LVN-PGE surfaces was 510 nM, with a linear range of 1–5 μM. Using the biosensor developed, DNA was detected within a linear range of 10–40 μg/mL, with a LOD of 2.74 μg/mL.

The development of a biosensor based on surface-enhanced Raman scattering (SERS) spectroscopy and electrochemical techniques was undertaken to explore the interaction between doxorubicin (DOX), a chemotherapeutic agent, and breast cancer DNA [16]. The electrochemical measurements revealed that the DNA–DOX interaction is dependent on DOX concentration. The study employed a reduced graphene oxide (rGO)-coated gold disk electrode (AuDE) decorated with plasmonic gold-coated (Fe_2_Ni@Au) magnetic nanoparticles, which were functionalized for the purposes of the investigation. DPV and CV were used to carry out the electrochemical measurements. These nanobiosensors have significant utility in analyzing the interactions between novel drugs and DNA.

Etoposide (ETO) is a highly effective clinical anticancer agent used to treat a variety of neoplastic diseases. As a semisynthetic podophyllotoxin derivative, ETO can be delivered by a range of nanomaterials due to its significant potential. The drug is commonly employed in the treatment of lung cancer, lymphoma, and leukemia, causing DNA damage in cancer cells and inhibiting tumor growth. In an earlier work [17], the electrochemical determination of ETO was investigated using a DNA biosensor developed through the utilization of DPV and square wave anodic stripping voltammetry. In that study, the fluorine tin oxide (FTO) electrode surface was coated with a graphene oxide–cobalt ferrite-ZnAl/layered double hydroxide composite, followed by DNA immobilization [17]. The developed biosensor achieved a LOD of 0.0010 μM for ETO.

**Figure 6:**
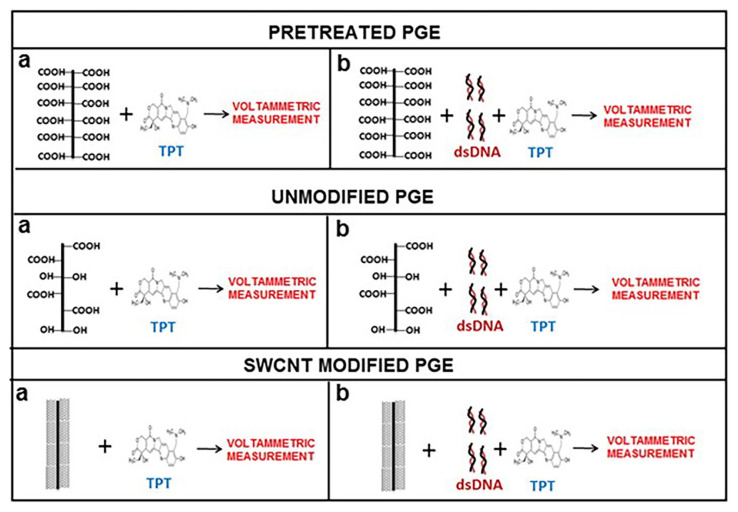
Detection of topotecan (TPT) (a) and the interaction between TPT and double stranded DNA (dsDNA) (b) at the surface of electrochemically pretreated pencil graphite electrodes (PGEs), unmodified PGEs and single walled carbon nanotube (SWCNT) modified PGEs by using DPV technique. Reprinted from Ref. [52], Copyright (2015), with permission from Elsevier.

In a study investigating the interaction between DNA and an anticancer drug, topotecan (TPT) (Figure 6), the TPT–DNA interaction was investigated for the first time using DPV and EIS [52]. The TPT molecule was detected with LODs of 0.51 and 0.37 μg/mL by using PGEs and single-walled carbon nanotube-modified PGEs (SWCNT-PGEs), respectively. A decrease in charge transfer resistance (R_ct_) values measured by EIS was observed as a result of the TPT–dsDNA interaction. The TPT–DNA interaction was explored based on changes in TPT and guanine signals.

There are also earlier studies in the literature based on the combination of electropolymerized redox active species and DNA, where the signal is attributed to changes in the redox status of the polymer [36,37,53–57].

Methylene blue (MB)-mediated detection of idarubicin, a chemotherapeutic drug, was developed using electrochemical methods [57]. It was reported that fsDNA was immobilized onto electropolymerized layers of azure B and, accordingly, the incorporation of MB into the DNA layers significantly increased the biosensor sensitivity [57].

In 2020, Porfirev et al. employed electrochemical methods for the first time to facilitate the physical adsorption of DNA molecules, and to conduct measurements of anthracycline derivative drugs, such as doxorubicin and daunorubicin, which are capable of intercalating with DNA. The ultimate goal of the study was to discover first the redox properties of proflavin polymers on a GCE, alongside the accumulation of DNA, employing diverse analytical techniques including CV, SEM, and EIS. The resultant findings revealed that while DNA exhibited a diminishing effect on peak currents in the voltammogram, it concurrently manifested an augmenting influence on charge transfer resistance [36].

An electrochemical DNA biosensor has been developed with the aim of accurately detecting anthracycline preparations. This biosensor is constructed based on an electropolymerized layer of neutral red (NR) and polycarboxyl-terminated thiacalix[4]arene-modified GCE. The intercalation of substances like doxorubicin, daunorubicin, and idarubicin into DNA results in an increase in charge transfer resistance, simultaneously reducing electron exchange and leading to a decrease in the cathodic peak of NR reduction [37].

#### 2.1.2 Nucleic acid biosensors for monitoring the interaction of DNA targeted molecules with nucleic acids

Nucleic acid biosensors can be designed to monitor the interaction of DNA-targeted molecules with nucleic acids. Nucleic acid biosensors are a powerful tool for monitoring the interactions of DNA-targeted molecules with nucleic acids. By providing a sensitive and selective means of detecting such interactions, these biosensors have the potential to accelerate the development of new drugs and diagnostic tools, as well as facilitating the study of fundamental biological processes.

An electrochemical biosensor was developed for the investigation of DNA interaction with the purine analog 6-TG used in the treatment of leukemia and lymphoma [58]. In the study, the PGE surface was modified with a single-walled carbon nanotube, and microscopic and electrochemical characterizations were performed with SEM and CV. The 6-TG and DNA interaction was investigated via DPV and EIS.

Mascini et al. reported a study presenting several antiproliferative metallodrugs interacting with dsDNA immobilized on SPEs [38]. They performed square wave voltammetry (SWV) in order to measure the guanine oxidation signal. It was reported that the interaction of cisplatin and carboplatin with DNA caused a decrease in the adenine oxidation signal, in contrast to little effect on the guanine oxidation signal. [Pt(bpy)(py)_2]_^2+^] was able to intercalate into dsDNA and produced a significant decrease in the oxidation signal of guanine, although not as much as cisplatin.

The study by Erdem et al. presented an assay in order to examine the interaction of cis-diamminedichloroplatinum(II) (cis-DDP) and cis-bis(3-aminoflavone)dichloroplatinum(II) (cis-BAFDP) with dsDNA using a PGE in combination with DPV [13]. The authors aimed to explore the drug–DNA interaction mechanisms by electrochemical method while determining the electrochemical and cytotoxic behavior of newly synthesized compounds. The results showed a decrease in guanine and adenine signals after cis-DDP interacted with dsDNA. In addition, a significant decrease was recorded in the signal of adenine after the interaction of cis-BAFDP with dsDNA.

An efficient electrochemical biosensor was developed by Song et al. to detect DNA damage resulting from the interaction of the antitumor agent AD-NU [59]. The detection was carried out using CV, DPV, and SWV. AD-NU was identified as the optimal ligand based on molecular docking analysis, which showed it had the minimum energy when binding to DNA or topoisomerase II (TOP II). To investigate the DNA damage caused by AD-NU, it is compared using the intensity changes of dGuo and dAdo oxidation peaks in DNA strands and monitored oxidative product 8-oxoGua.

The studies on the detection of DNA interactions with anticancer drugs or drug candidates are summarized in Table 1.

### 2.2 The interaction of central nervous system drugs/candidate drugs with DNA

The interaction between central nervous system drugs or potential drugs and DNA is an important area of research. It is known that various drugs used in the treatment of neurological disorders, such as antipsychotics, antidepressants, anxiolytics, and antiepileptics, interact with DNA and cause changes in the structural and chemical stability of this interaction. The interaction between drugs and DNA has important effects on the pharmacological activity and toxicity of drugs. Techniques such as electrochemical methods, fluorescence spectroscopy, and molecular docking analysis are used to investigate drug binding to DNA and to study the effect of drug–DNA interactions on the structure and function of DNA. The interaction between central nervous system drugs and DNA is of key importance to understanding the drug mechanisms of action and possible side effects. Therefore, the study of the interaction between central nervous system drugs and DNA plays a role in developing safe and effective drugs or drug candidates for the treatment of neurological disorders. The recent studies on the interaction between central nervous system drugs/candidate drugs and DNA are summarized in Table 2.

Investigation of drug–DNA interactions is a promising approach for discovering new compounds for biochemical target analyzing and DNA biosensor design [18]. An electrochemical ct-dsDNA biosensor was developed for detecting the interaction between the drug aripiprazole (ARP) and dsDNA [18]. The ctdsDNA-modified GCE detected the changes in the ARP and DNA bases signals caused by the interaction with ctdsDNA. The interaction studies conducted in the solution phase confirmed the changes in the DNA bases signal. The investigation revealed a correlation between the results of electrochemistry and UV-Vis spectrophotometry, as both techniques detected a decline in ARP signal. This study highlights the usefulness of voltammetric approaches and electrochemical DNA-based biosensors in the investigation of DNA–drug interactions in a straightforward and efficient manner.

Vaníčková et al. examined the enzymatic degradation of a potential local anesthetic drug using a DNA-based electrochemical biosensor with a screen-printed electrode [19]. They used DPV to monitor the changes in drug concentration in blood serum. The researchers developed a method for determining the drug concentration in blood serum through protein precipitation and electrochemical measurements with the DNA biosensor, and attempted to use it to evaluate the drug’s enzymatic degradation in human and rabbit sera. The study demonstrated that a DNA-based biosensor with high sensitivity can effectively evaluate the degradation of the drug.

Ensafi et al. developed an electrochemical biosensor for studying the interaction between codeine and morphine with dsDNA at pH 7.0 [101]. The dispersant of multiwalled carbon nanotubes (MWCNTs) was poly(diallyldimethylammonium chloride) (PDDA). DPV was employed at a PGE to electrochemically oxidize both molecules due to the presence of phenolic and amino groups in their structures. The biosensor was successfully used for analyzing codeine and morphine in blood serum, urine samples, and pharmaceutical formulations, indicating its potential for practical applications. Asghary et al. utilized voltammetric techniques to investigate the electrochemical oxidation of ketamine, an anesthetic and analgesic drug, at a CPE [102]. The authors examined the interaction between ketamine and DNA and found that ketamine demonstrated a specific affinity towards guanine bases of DNA. The study utilized differential pulse voltammetry and UV-Vis spectroscopy to demonstrate the interaction. The authors successfully implemented the proposed method for the voltammetric determination of ketamine in real samples, including serum samples, as demonstrated by DPV. Nimal et al. conducted a study aimed at investigating the interaction between paroxetine, a phenylpiperidine derivative drug that functions as an effective serotonin reuptake inhibitor, and biomolecules using electrochemical and fluorescence spectroscopy, and molecular docking [103]. The interaction between paroxetine and biomolecules was investigated using DPV, which showed a reduction in the anodic oxidation signal of deoxyguanosine in ctdsDNA, indicating the interaction. In addition to electrochemical and fluorescence spectroscopy studies, molecular docking analysis was employed to elucidate the interaction between paroxetine and ctdsDNA.

### 2.3 The interaction of antibiotic drugs/candidate drugs with DNA

Antibiotics are drugs used to treat infections caused by microorganisms such as bacteria, fungi, and parasites. These drugs work by either disrupting the structure of the cell wall of the microorganism or inhibiting its ability to reproduce. Bactericidal antibiotics directly kill bacteria, while bacteriostatic antibiotics slow or stop the growth of bacteria [104]. Bactericidal drugs have a stronger effect in killing bacteria compared to bacteriostatic drugs, which only slow down or inhibit the growth and reproduction of bacteria. However, some bacteriostatic drugs can have a bactericidal effect when used in combination. Bacteriostatics are more suitable for patients with a strong immune system, as their body’s immune system can eliminate the bacteria that have been slowed down. Bactericides, on the other hand, are used when the immune system is compromised and weak, as they directly kill the bacteria [105]. The recent studies of different antibiotic drugs/potential drugs such as ciprofloxacin, isoniazid, levofloxacin, and nitrofurantoin will be discussed in this section. There are numerous studies in the literature focused on the investigation on the DNA interaction of antibiotic drug/candidate drugs (Table 3).

The effect of 2-(1H-benzimidazol-2-yl) phenol (2-Bip), a benzimidazole derivative, with sequence-specific DNA was investigated by DPV in combination with a PGE [106]. The researchers used electrochemical methods to examine the changes in both guanine and 2-Bip oxidation signals. In the study, the impact of 2-Bip on single-stranded DNA (ssDNA) and dsDNA was assessed. The results revealed that 2-Bip exhibited moderate toxicity to ssDNA and significant toxicity to dsDNA. The study postulated that the interaction between 2-Bip and DNA possibly involved noncovalent bonds. In addition, it was suggested that 2-Bip could serve as a novel DNA hybridization indicator due to its distinct effects on ssDNA and dsDNA, and that it has the potential to be utilized as a drug molecule due to its impact on DNA.

Ciprofloxacin is an antibiotic drug used for the treatment of various bacterial infections, such as respiratory tract infections, urinary tract infections, skin infections, gastrointestinal infections, and other bacterial infections [107]. It works by stopping or killing the growth of bacteria to help fight the infection. The mechanism of ciprofloxacin’s effect on DNA was studied by Nawaz et al. using an electrochemical nucleic acid biosensor [107]. They employed potentiometry and voltammetry to examine the interaction between ciprofloxacin and DNA at a GCE. Additionally, they investigated the influence of sodium and calcium ions on this interaction under different solution conditions to better understand the underlying mechanism. In another study for the determination of ciprofloxacin, a DNA biosensor for ciprofloxacin detection was developed by Cheraghi et al., using a PGE modified with polypyrrole, single-wall carbon nanotubes, and dsDNA (PGE/PP/SWCNTs/DNA) [108]. It is capable of analyzing drug and urine samples. Diab et al. developed an electrochemical biosensor and examined the voltammetric behavior of ciprofloxacin using voltammetric techniques with a GCE [109]. Additionally, the interaction between ciprofloxacin and DNA was studied using CV.

Isoniazid (INZ) is an antibiotic used for the treatment of tuberculosis, an infection caused by the bacterium *Mycobacterium tuberculosis* [110]. The drug works by inhibiting the growth and multiplication of bacteria, thus helping to control the infection. The development of a new DNA sensor for the detection of INZ was reported by Moghaddam et al., using carbon/La^3+^/CuO (C/La^3+^/CuO) nanocomposites [110]. The sensor was modified with C/La^3+^/CuO to enhance electrical conductivity and surface area. The biosensor was successfully used to detect INZ in serum and urine samples, making it a promising platform for effective electrochemical analysis.

Levofloxacin is an antibiotic drug used to treat bacterial infections such as respiratory tract infections, urinary tract infections, skin infections, sinusitis, and pneumonia. The levofloxacin and dsDNA interaction in serum and urine samples was detected using an electrochemical sensor developed by Radi et al. using a GCE and CPE [39]. The optimization studies such as on accumulation time and drug concentration were carried out using CV. The LOD was 1.0 × 10^−7^ M in the range of 5.0 × 10^−7^ to 5.0 × 10^−6^ M in serum, whereas it was 25 μg/mL of levofloxacin in the urine sample. Electrostatic binding and intercalation were identified as the binding model of levofloxacin to DNA under the conditions of the study.

Nitrofurantoin is a type of antibiotic used for treating bacterial infections by hampering the bacterial ability to reproduce. Aydoğdu et al. performed a study to electrochemically monitor, for the first time, the interaction between nitrofurantoin (NFT) and dsDNA using a poly(5-amino-2-mercapto-1,3,4-thiadiazole) (PAMT)-modified GCE [111]. The GCE/PAMT/dsDNA electrode was used by immobilizing dsDNA on PAMT deposited on the GCE. DPV was employed to examine the NFT–dsDNA interaction. The results demonstrated that this DNA biosensor could accurately and precisely determine the NFT–dsDNA interaction within a linear concentration range of 2–25 mg/L in human serum samples.

Li et al. utilized gold nanoparticle-modified Au electrodes to study the DNA-specific binding properties of natural molecules such as nogalamycin, mithramycin, and netropsin [112]. The gold nanoparticle modification resulted in a 20- to 40-fold enhancement in the detection limit. The specificity of interactions of these molecules with DNA was compared via EIS in a redox probe on GC-rich DNA-modified substrate and AT-rich DNA-modified substrate. Mithramycin, a G-C specific-DNA binding anticancer drug; netropsin, an A-T specific-DNA binding drug; and nogalamycin, an intercalator, were used as the model molecules.

Rifampicin (RF) is an antibiotic used for the treatment of bacterial infections. Shumyantseva et al. investigated the electrochemical interactions of dsDNA and RF or and RF encapsulated in phospholipid micelles (nanosome/RF) (NRF) [113]. The findings have implications for the development and optimization of drug delivery systems containing DNA-targeted molecules to use in chemotherapy, diagnostics, and therapeutic effectiveness.

### 2.4 The interaction of antiviral drugs/candidate drugs with DNA

Antiviral drugs are pharmacological agents used against viral infections. They are employed to control or eradicate viral infection. In general, viral medications are typically delivered through intravenous administration rather than oral administration. This is because intravenous delivery allows more rapid and potent drug action within the bloodstream. However, it is essential to carefully monitor the dosage of antiviral medications to prevent overuse, which could lead to the development of drug resistance and decreased efficacy in treating other viral infections [114]. This section summarizes the electrochemical detection of the interaction of DNA with some drugs that have been commonly used as antiviral drugs such as didanosine (DDI), lycorine (LYC), nitazoxanide (NTZ), and tenofovir (*tnf*) (Table 4).

DDI is a reverse transcriptase inhibitor drug used in the treatment of HIV/AIDS and is important in the determination of biological samples. Despite its efficacy, it has side effects such as nausea, vomiting, stomach pain, tingling, burning, and numbness. Karimi-Maleh et al. developed a sensitive and cost-effective DNA biosensor for the determination of DDI using voltammetry. The team modified the surface of a PGE with a polypyrrole (PPy) and reduced graphene oxide (rGO) composite to enhance the sensitivity of the biosensor [115]. EIS and SEM were employed in the characterization processes of the developed biosensor, while DPV was used to investigate the interaction between DDI and dsDNA. The developed PPy/rGO/PGE immobilized with dsDNA biosensor was able to accurately and selectively determine DDI in real samples.

LYC is a compound with notable antiviral effects against poliomyelitis, coxsackie, and herpes type 1 viruses. A PGE was used to study the interaction between LYC and calf thymus ssDNA while observing a decrease in guanine and adenine signals using DPV [116].

NTZ is a broad-spectrum antiparasitic and antiviral drug used to treat various helminthic, protozoal, and viral infections [117]. Radi et al. investigated the interaction of NTZ with salmon sperm double-stranded deoxyribonucleic acid (ss-dsDNA) using UV-Vis spectroscopy, CV, and DPV and observed that its electrochemical behavior and spectral properties changed after the NTZ and ss-dsDNA interaction [20].

Finally, *tnf* is an antiviral drug used to treat, prevent, and manage HIV infection, but can cause kidney and liver problems as serious side effects [118]. Morawska et al. investigated the interaction of *tnf* with DNA using electrochemical and spectroscopic techniques, and utilized a boron-doped diamond electrode (BDDE) to investigate the electrochemical behavior of the *tnf*–DNA interaction using SWV [21].

### 2.5 The interaction of other drugs/candidate drugs with DNA

This section summarizes the electrochemical detection of the interaction of DNA with other drugs that have been commonly used as potential drugs, such as benznidazole (BZN), capsaicin (CPS), metformin (MET), nifedipine (NDP), lercanidipine (LDP), and amlodipine (AMP) (Table 5).

**Figure 7:**
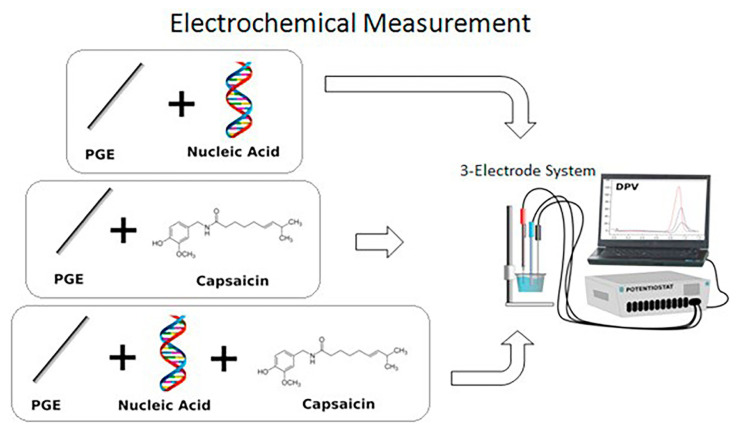
Experimental presentation for the immobilization of CPS and nucleic acids at the surface of PGEs, and electrochemical detection of interaction between CPS and nucleic acids at the surface of PGEs. Reprinted from Ref. [120], Copyright (2017), with permission from Elsevier.

Capsaicin (trans-8-methyl-N-vanillyl-6-nonenamide) (CPS) is the active compound found in capsicum and is reported to have anticarcinogenic properties. It is an irritant and neurotoxin chemical for mammals, including humans, and produces a burning sensation in any tissue it comes into contact with. The interaction between DNA and CPS was investigated using PGE and DPV as shown in Figure 7 [120]. In addition, voltammetric determination of the interaction between CPS and dsDNA, cDNA from Huh7 human hepatocellular carcinoma cell line, and total RNA isolated from PCR samples was performed and electrophoretic studies were also conducted in order to confirm their results.

Metformin (MET) is an orally administered antihyperglycemic drug used to control type II diabetes. It is a first-line treatment option for patients who are overweight, obese, or have normal renal function due to its cholesterol and triglyceride-lowering effects. Machini et al. developed a method for the detection of MET, which is considered an important contaminant in wastewater [22]. In this method, they investigated the electrochemical behavior of MET using a carbon black dihexadecylphosphate film-modified electrode. They took advantage of the catalytic effect of Cu(II) ions on the oxidation of MET by using the MET–Cu(II) complex, which resulted in an increase in current signal. The method was performed using techniques such as DPV and SWV. The electroanalytical LOD for the MET–Cu(II) complex was 0.63 μM, with a limit of quantitation of 2.09 μM.

Calcium channel blockers, also known as calcium antagonists, are medications that are used to treat various health conditions including high blood pressure, angina, heart rhythm disorders, and circulatory disorders. These drugs function by decreasing the amount of calcium that enters the cells of the heart and smooth muscles. This results in the relaxation and widening of blood vessels, which leads to improved oxygen supply to the heart and a decrease in blood pressure. NDP, LDP, and AMP are types of calcium channel blockers that belong to a subgroup called dihydropyridines. Shahzad et al. studied the interaction of these three calcium antagonists with calf thymus dsDNA [122]. The changes in response were monitored and accordingly decreases in guanine and adenine peak currents were recorded while indicating a possible interaction between the molecules and DNA. Using various methods such as electrochemical techniques, UV-Vis absorption spectroscopy, and molecular analysis, they investigated how NDP and LDP bind to ctDNA through hydrogen bonds and van der Waals forces. They also used DPV and molecular analysis to investigate the interaction between AMP and dsDNA. They reported a decrease in guanine and adenine peak currents since NDP and LDP interact with dsDNA.

Theophylline (TP) is a methylxanthine alkaloid that can be found in various food sources such as cocoa beans, black tea, and, to a lesser extent coffee and chocolate. This compound has stronger effects on heart and respiration compared to caffeine and is therefore used as a medication to treat cardiovascular and respiratory diseases, particularly chronic obstructive pulmonary disease [125]. To investigate the interaction and binding mode of TP with low molecular salmon sperm DNA, ssDNA, and mononucleotides, Nemčeková et al. developed a DNA-based biosensor [23]. The association of theophylline with nucleobases was confirmed by UV-Vis and FTIR spectra, and the structure of the DNA–theophylline complex was predicted based on hydrogen bonding.

Benznidazole (BZN) is a medication used to treat a parasitic infection called Chagas disease. BZN is usually taken by mouth and the duration of the treatment varies depending on the severity of the infection. By killing the parasite, this medication helps to alleviate the symptoms associated with Chagas disease [126]. La-Scalea et al. have used an electrochemical technique to detect and measure the damage caused by reduced benznidazole in DNA [119]. Based on their results they proposed to provide real-time data on the extent of damage to DNA and offered a way to screen for potential damage to DNA structure.

## 3. Conclusion

DNA plays a fundamental role in critical cellular processes such as transcription, translation, and replication, making it a primary target for numerous clinically utilized pharmaceuticals, including antivirals, anticancer drugs, and antibiotics. Small molecules have the ability to interact with the DNA helix, and such interactions can impede or modify DNA functioning. Various types of drug–DNA interaction exist, including intercalation, noncovalent interaction, cross-linking, nucleoside–analog binding, and DNA cleavage. Each type of interaction operates through a distinct mechanism. Investigating drug–DNA interactions is an essential process not only for understanding the underlying mechanisms, but also for obtaining significant knowledge to aid in the design of effective drugs. DNA serves as the primary target for numerous clinically relevant pharmaceuticals, including antiviral, anticancer, and antibacterial agents.

Electrochemical biosensors have proven to be an effective tool for monitoring drug–DNA interactions. They offer numerous advantages such as high sensitivity, selectivity, simplicity, and portability, making them ideal for use in both research and clinical settings. These biosensors rely on the detection of changes in electrochemical signals that occur as a result of interactions between drugs and DNA.

In addition, the development of new materials and innovative biosensing strategies has significantly enhanced the performance of electrochemical biosensors, making them even more useful for drug–DNA interaction studies. The use of nanomaterials in biosensor design has many advantages. Nanomaterials have a high surface area-to-volume ratio. This advantage can yield more nucleic acids immobilized onto the electrode surface while resulting in efficiency of the interaction of nucleic acids with drug molecules. It also provides an increased sensitivity of biosensor with lower detection limits. Nanomaterials are generally intrinsically stable and resistant to degradation. The use of nanomaterials in biosensors increases their durability and long-term performance. Since some of these nanomaterials are biocompatible, they can be preferentially used more in biosensor design. They are also important for drug development studies, especially for those investigating DNA’s interaction with a DNA-targeted drug. With the advances in nanotechnology and materials science, it is thought that the performance and diversity of biosensors developed for drug–DNA interactions will increase in the future. However, there are still some challenges that need to be addressed, such as improving the stability and reproducibility of the biosensors and expanding their application to a wider range of drugs and DNA targets.

Electrochemical biosensors show great promise for the monitoring of drug–DNA interactions, and will continue to be an important area of research in the future. The ongoing development of new materials and innovative biosensing strategies is expected to further enhance the performance of these biosensors, making them even more useful for drug–DNA interaction studies in both research and clinical settings.

## Figures and Tables

**Table 1:** Electrochemical biosensors for the interactions of DNA with anticancer drugs/drug candidates.

DRUG/DRUG CANDIDATE	ELECTRODE	METHOD	LINEAR RANGE	LIMIT OF DETECTION (LOD)	APPLICATION	REFERENCE
4,4′-Dihydroxy chalcone (DHC)	dsDNA-modified CPE	DPV	-	63 nM for dsDNA, 42 nM for ssDNA	DHC solution	[60]
5-Fluorouracil (5-FU)	GCE/P(BCP)/dsDNA	DPV	1.0–50 mg/L	0.31 mg/L	5-FU solution	[61]
6-Thioguanine (6-TG)	dsDNA-modified PGE	DPV	20–80 μg/mL	4.60 μg/mL	6-TG solution	[51]
	SWCNT-modified PGE	DPV	1–2.5 μM	0.25 μM	6-TG solution	[58]
N-(2-chloroethyl)-N-nitrosourea (AD-NU)	CtDNA/GCE	CV, DPV, and SWV	-	-	AD-NU solution	[59]
Adriamycin	dsDNA onto GCE	DPV	-	-	Adriamycin solution	[62]
Amsacrine	ds-DNA/Eu^3+^-doped NiO/CPE	DPV	0.1–100 μM	0.05 μM	Human serum and urine	[44]
Azacytidine	dsDNA/GCE	DPV	20–70 μM	Guanine: 9.81 μM Adenine: 1.34 μM	Azacytidine solution	[32]
Bevacizumab (BEVA)	dsDNA/GCE	DPV	-	-	BEVA solution	[63]
Bleomycin (BLM)	dsDNA/ZnSB/AuNPs/PGE	EIS and DPV	-	-	BLM solution	[64]
*cis*-Bis(3-aminoflavone)dichloroplatinum(II) (*cis-*BAFDP)	dsDNA-modified PGE	DPV	-	600 nM	-	[13]
Cisplatin	EDTA metallothionein-modified HMDE	DPV and CV	2–1000 nM	100 pM	Cisplatin solution	[65]
Cisplatin, Carboplatin, [Pt(bpy)(py)2][PF6]2, Titanocene dichloride TiCp_2_Cl_2_, NAMI-A)	ds-DNA-modified SPE	SWV	0–1.0 mM	-	-	[38]
Cloforabine (CLF)	dsDNA-modified GCE	DPV	1.6 and 25.3 μM	0.08 μM	Serum samples	[66]
Cyclophosphamide	CNT-modified SPE	DPV	-	1 μM	Cyclophosphamide solutions	[67]
Cytarabine (CBN)	dsDNA/Eu^3+/^Cu_2_O CLFNs/CPE	DPV	0.01 and 90 μM	2.8 nM	Human serum	[68]
Daunorubicin (DNR)	PGE modified with cQD	DPV and EIS	1.17–0.89 μg/mL	DNR: 0.02 μg/mL ctDNA: 0.89 μg/mL	DNR solution	[31]
	NFs-modified PGE	DPV	5–20 μM	c-NFs-PGE: 2.93 μM ga-NFs-PGE: 2.06 μM	DNR solution	[46]
	rGO-modified PGE	DPV	5–25 μM	0.55 μM	DNR solution	[69]
	LVN-PGE	DPV and CV	10–40 μg/mL	2.74 μg/mL	DNR solution	[35]
	CHIT-PGE	DPV	2.50–10 μg/mL	0.60 μM	DNR solution	[70]
Doxorubicin (DOX)	AuDE/SAM/ rGO/Fe_2_Ni@Au/dsDNA	DPV	0.003–5 mg/mL	8 μg/mL	Real biological DNA	[16]
	GCE with OLA-cone and polymers, PEI, and DNA	CV	20–200 ng/L	5 ng/L	Real sample (milk sample)	[71]
	Pt/MWCNTs	CV	0.05–4.0 μg/mL	0.002 μg/mL	Blood sample	[72]
Echinomycin (Echi)	DNA-modified HMDE	CV	-	-	Echi solution	[15]
Ellipticine, doxorubicin, Ethidium bromide	Screen-printed nanotube carbon electrodes, HMDE, dsDNA	SWV and CV	-	-	Biological samples	[73]
Epirubicin (EPR)	ssDNA-modified and dsDNA-modified CPE	DPV and CV	-	0.62 μM	EPR solution	[24]
	AuNCs/GSPE	DPV	0.04–0.80 μM and 0.8–20.0 μM	0.01 μM	Human blood serum	[45]
Etoposide (ETO)	GO/CoFe_2_O_4_/ZnAl-LDH/FTO	DPV	0.2–10 μM	0.0010 μM	Human blood plasma, serum, and urine	[17]
Fludarabine	dsDNA/GCE	DPV	1.6–14.8 μM	0.28 μM	Biological samples	[74]
Flutamide (Flu)	HEM/dsDNA/MCM41/SPE HEM/ssDNA/MCM41/SPE	DPV	0.7–10 mM	0.1 μM	Human serum	[75]
Ganciclovir (GCV)	Fe_3_O_4_/cMWCNTs/GCE	SWV	0.08–53 μM	20 nM	Blood serum and human urine	[76]
Gemcitabine (GEM)	MWCNTs/Ag NPs/dsDNA MIS film-modified CPE	DPV	1.5–93 μM	Guanine: 12.5 nmol/L adenine: 48.8 nmol/L	Human serum	[77]
	dsDNA/GCE	DPV and SWV	-	-	GEM solution	[78]
	GCE/P(PDCA)/dsDNA	DPV	1–30 mg/L	0.276 mg/L	Human serum	[79]
Idarubicin (IDA)	dsDNA/GCE	CV and DPV	-	-	IDA solution	[80]
	ctdsDNA/PGE	DPV	60–80 μg/mL	-	IDR solution	[81]
	Pt–Pd–ZnO/SWCNT-modified GCE	DPV	1.0 nM–65 μM	0.8 nM	Serum sample	[33]
	PP/La_2_O_3_NP@SF-L Cu_2_S NS/PGE	DPV	0.01–500.0 μM	1.3 nM	Human blood serum	[82]
Irinotecan (CPT-11)	poly(CTAB-MWCNTs)/PGE	DPV	4–150 μg/mL	3.06 μg/mL	Serum	[83]
Lapatinib (LPT)	ctdsDNA/GCE	CV, DPV, SWV	-	-	LPT solution	[84]
Leuprolide (LPR)	dsDNA-modified PGE	DPV	0.20–1.00 ppm	0.04 ppm	Plasma sample	[85]
Methotrexate (MTX)	dsDNA/GCE	DPV	-	-	MTX–DNA solutions	[86]
	dsDNA/GCE	SWV	2.0 × 10^−^^8^ –4.0 × 10^−^^6^ mol/L	5.0 × 10^−^^9^ mol/L	Urine samples	[87]
Mitomycin C (MC)	dsDNA-modified PGE	DPV	0.1–10 μg/mL	0.23 μg/mL	MC solution	[88]
	SWCNT/PVF^+^-modified PGE	DPV	2.5 to 40 μg/mL	625 ng/mL	MC solution	[89]
	MWCNT/CHIT/PGE	DPV	10 to 50 μg/mL	1.12 μg/mL	MC solution	[47]
	CHIT-IL PGE	DPV	10 to 140 μg/mL	4.47 mg/mL	MC solution	[48]
	L-lysine NFs glycine NFs	DPV	20–100 μg/mL	MC: 12.55 μg/mL	MC solution	[34]
	Chitosan-CNT/PGE	DPV	5–50 μg/mL	MC: 11.01 μg/mL	MC solution	[14]
Mitoxantrone (MTX)	dsDNA-coated MWCNT-Ag-PT GCE	DPV	0.05–100.0 μM	13 nM	Urine and serum	[49]
	Calomel electrode modified with MWCNT	Potentiometry	1–1000 ng/mL	150 ng/mL	MTX solution	[90]
N-(2-(naphthalimide)ethyl)-2-(2-nitroimidazole) acetamide (D-2) N-(2-(5-nitro-)naphthalimide)ethyl)-2-(2-nitroimidazole) acetamide (D-5)	ctDNA/GCE	CV and DPV	-	-	-	[91]
Nilotinib	CPE modified with In^3+^/NiO RLHNS	DPV	0.01–50.0 μM	0.62 nM	Serum sample	[92]
Nivolumab (NIVO)	dsDNA-modified GCE	CV, DPV, and EIS	-	-	NIVO solution	[50]
Taxol	Au electrode modified with azidohexane thiol derivative as a self-assembled monolayer (SAM)	CV and EIS	1.2 × 10^−^^7^–5 × 10^−^^6^ M	1.2 × 10^−^^8^ M	Human sample serum	[93]
	dsDNA-MWNTs-TiO_2_/ZrO_2_–CHIT-PGE	DPV	0.7–1874.0 nmol/L	0.01 nmol/L	Serum and urine	[94]
Temodal	dsDNA/Au-NPs/PGE	DPV	5.0 nM–45.0 μM	1.0 nM	Capsule, urine, and blood serum	[95]
Temozolomide (TMZ)	ssDNA and dsDNA/PGE	DPV	10–1000 μg/mL	250 μg/mL	TMZ solution	[96]
Topotecan (TPT)	SWCNT/PGE	DPV	0.05 and 10 μg/mL	0.51 μg/mL	TPT solution	[52]
	DNA-immobilized SPE	EIS	-	-	TPT solution	[97]
	dsDNA/GPE	DPV	0.7 to 90.0 μM	0.37 μM	Human blood serum and urine	[98]
Valrubicin	AuNPs/en/MWCNTs/Au electrode	CV	0.5–80.0 μmol/L	0.018 μmol/L	Human urine and blood serum	[99]
VP-16	MWCNTs/PEOY/GCE	CV	3.0 × 10^−^^8^–3 × 10^−^^7^ M	0.836 nM	-	[100]

**Abbreviations:** Modification: dsDNA: double-stranded DNA, *ct*DNA*: calf thymus* DNA, ssDNA: single-stranded DNA, ctdsDNA: calf thymus double-stranded DNA. P(BCP): Poly(bromocresol purple, Eu^3+^: europium (III), AuNPs: gold nanoparticles, ZnSB: zinc(II)-Schiff base complex, CNT: carbon nanotube, Eu^3+^/Cu_2_O CLFNs: Eu^3+/^Cu_2_O nanostructures with clover-like faces, Cu_2_O: cuprous oxide, cQD: carbon quantum dot, NFs: Nanoflowers, rGO: reduced graphene oxide, LVN: Levan, CHIT: Chitosan, PEI: poly(ethylene imine), MWCNTs: multiwalled carbon nanotubes, Pt: platinum, HMDE: hanging mercury drop electrode, AuNCs: gold nanocubes, LDHs: layered double hydroxides, CoFe_2_O_4_: cobalt ferrite, GO: graphene oxide, MCM41: Mobil Composition of Matter, HEM: Hemin, cMWNTs: carboxylated multiwalled carbon nanotubes, P(PDCA): poly(2,6-pyridinedicarboxylic acid), SWCNT: single-wall carbon nanotubes, Cu_2_S: Copper sulfide, PP: Polypyrrole, SF: snowflake, La_2_O_3_: Lanthanum oxide, poly(CTAB-MWCNTs): cetyl trimethylammonium bromide-multiwalled carbon nanotubes, PVF+: poly(vinylferrocenium), PT: polythiophene, In^3+^/NiO RLHNSs: raspberry-like indium(III)/nickel oxide hierarchical nanostructures, PEOY: poly Eosin-Y. Electrode: CPE: carbon paste electrode, PGE: pencil graphite electrode, GCE: glassy carbon electrode, GSPE: graphite screen-printed electrode, FTO: fluorine tin oxide, GPE: graphene paste electrode, AuE: Au electrode. Method: DPV: differential pulse voltammetry, SWV: square wave voltammetry, CV: cyclic voltammetry, EIS: electrochemical impedance spectroscopy

**Table 2:** Electrochemical biosensors for the interaction of DNA with central nervous system drugs/drug candidates.

DRUG/DRUG CANDIDATE	ELECTRODE	METHOD	LINEAR RANGE	LIMIT OF DETECTION (LOD)	APPLICATION	REFERENCE
1-Methyl-2-piperidinoethylester of 2-hexoxyphenylcarbamic	dsDNA-modified SPE	DPV	0.013–0.200 μM for human blood serum, 0.05–1.00 μM for rabbit blood serum	0.06 μM for rabbit blood serum, 0.016 μM for human blood serum	Blood serum matrix	[19]
Aripiprazole (ARP)	ctdsDNA/GCE	DPV	4.50 × 10^−^^7^ M and 1.12 × 10^−^^6^ M	1.12 × 10^−^^6^ M	ARP solution	[18]
Codeine and morphine	dsDNA/MWCNTs–PDDA/PGE	DPV	Codeine: 0.05–40 μg/mL Morphine: 0.05–42 μg/mL	Codeine: 0.041 μg/mL Morphine: 0.043 μg/mL	Blood serum and urine	[101]
Ketamine	ssDNA/CPE	DPV	0.0–6.0 × 10^−7^ M	1.98 nM	Serum	[102]
Paroxetine (PARO)	ctdsDNA/GCE	DPV	1–50 μM	0.29 μM	Urine	[103]

**Abbreviations:** Modification: ssDNA: single-strand DNA, dsDNA: double-strand DNA, ctdsDNA: calf-thymus double strand DNA, MWCNT: MWCNT: multiwalled carbon nanotubes, PDDA: Poly(diallyldimethylammonium chloride), Electrode: PGE: pencil graphite electrode, GCE: glassy carbon electrode, CPE: carbon paste electrode, SPE: screen-printed electrode, Method: DPV: differential pulse voltammetry.

**Table 3:** Electrochemical biosensors for the interaction of DNA with antibiotic drugs/drug candidates.

DRUG/DRUG CANDIDATE	ELECTRODE	METHOD	LINEAR RANGE	LIMIT OF DETECTION (LOD)	APPLICATION	REFERENCE
2-(1H-benzimidazol-2-yl) Phenol	dsDNA and ssDNA/PGE	DPV	-	4.2 μmol/L	2-Bip solution	[106]
Ciprofloxacin	dsDNA-modified GCE	DPV	40–80 μM	24 μM	-	[107]
	PGE/PP/SWCNTs/DNA	DPV	0.008–30.0 μM	4.0 nM	Drug and urine samples	[108]
	DNA/GCE	DPV	1.0–10.0 μM	0.117 μM	Ciprofloxacin solution	[109]
Isoniazid (INZ)	C/La^3+/^CuO/CPE	DPV	1.0–165.0 μM	0.035 μM	Serum and urine samples	[110]
Levofloxacin	GCE and carbon paste electrode	CV	5.0 × 10^7^–5.0 × 10^−^^6^ M	1.0 × 10^−^^7^ M	Urine sample	[39]
Nitrofurantoin (NFT)	GCE/PAMT/dsDNA	DPV	2–25 mg/L	0.65 mg/L	Human serum	[111]
Nogalamycin, Mythramycin, Netropsin	Gold nanoparticles-modified Au electrode	CV, EIS	Mithramycin: 15 nM–1 μM netropsin: 40 nM–1 μM	Nogalamycin: 5 nM mythramycin: 10 nM netropsin: 40 nM	-	[112]
Rifampicin (RP) and Nanosome/Rifampicin	SPE/PB_290_-b-PDMAEMA_240_/MWCNT	DPV	20–600 μM	1.5 mg/mL	RF solutions	[113]

**Abbreviations:** Modification: ssDNA: single-strand DNA, dsDNA: double-strand DNA, PP: polypyrrole, SWCNT: single-wall carbon nanotubes, C: carbon, PAMT: poly(5-amino-2-mercapto-1,3,4-thiadiazole, PB_290_-b-PDMAEMA_240_: poly(1,2-butadiene)-block-poly(2-(dimethylamino)ethyl methacrylate), MWCNT: multiwalled carbon nanotubes, Electrode: PGE: pencil graphite electrode, GCE: glassy carbon electrode, CPE: carbon paste electrode, SPE: screen printed electrode, Method: DPV: differential pulse voltammetry, CV: cyclic voltammetry, EIS: electrochemical impedance spectroscopy.

**Table 4:** Electrochemical biosensors for the interaction of DNA with antiviral drugs/drug candidates.

DRUG/DRUG CANDIDATE	ELECTRODE	METHOD	LINEAR RANGE	LIMIT OF DETECTION (LOD)	APPLICATION	REFERENCE
Didanosine (DDI)	PGE/PPy/rGO	DPV	0.02–50.0 μM	8.0 nM	Urine sample	[115]
Lycorine (LYC)	dsDNA-modified CPE and PGE	DPV	-	30.2 ng/mL	-	[116]
Nitazoxanide (NTZ)	DNA/SPCE	DPV	0.1–25.0 μM	0.025 μM	Serum	[20]
Tenofovir (*tnf*)	BDDE/dsDNA, ssDNA	SWV	5.0 × 10^−^^6^ – 1.0 × 10^−^^4^ mol/L	5.6 × 10^−^^7^ and 1.9 × 10^−^^6^ mol/L	*tnf* standard solution	[21]

**Abbreviations:** Modification: ssDNA: single-strand DNA, dsDNA: double-strand DNA, PPy: polypyrrole, rGO: reduced graphene oxide, Electrode: CPE: carbon paste electrode, PGE: pencil graphite electrode, SPCE: screen-printed carbon electrode, BDDE: boron-doped diamond electrode, Method: DPV: differential pulse voltammetry, SWV: square wave voltammetry

**Table 5:** Electrochemical biosensors for the interaction of DNA with other drugs/drug candidates.

DRUG/DRUG CANDIDATE	ELECTRODE	METHOD	LINEAR RANGE	LIMIT OF DETECTION (LOD)	APPLICATION	REFERENCE
Benznidazole (BZN)	ssDNA-modified GCE	DPV	-	1.0 μM	-	[119]
Capsaicin (CPS)	chitosan-SWCNT composite-modified PGE	DPV	1–5 μg/mL	0.62 μg/mL	CPS with PCR samples	[120]
Furazolidone (FU)	dsDNA/TiO2@rGO-CPE	DPV	1.0–150.0 pmol/L	Guanine: 0.55 pmol/L Adenine: 0.43 pmol/L	Blood serum and urine	[121]
Metformin (MET)	CB−DHP/GCE	DPV	-	0.63 μM	Wastewater sample	[22]
Nifedipine (NDP), lercanidipine (LDP), and amlodipine (AMP)	dsDNA/GCE	CV	-	-	-	[122]
Pyrimethamine	ss-dsDNA/SPCE	DPV	1.0 × 10^−^^7^ to 5.0 × 10^−^^5^ M	1.0 × 10^−^^8^ M	Human serum samples	[123]
Quinacrine	DNA–Cu(II)/PAA/GCE	Amperometry	-	10 μM	Quinacrine solution	[124]
Theophylline (TP)	dsDNA/GCE	SWV	-	-	TP solution	[23]

**Abbreviations:** Modification: ssDNA: single-strand DNA, dsDNA: double-strand DNA, SWCNT: single-wall carbon nanotube, rGO: reduced graphene oxide, CB−DHP: carbon black dihexadecylphosphate film, PAA: polyallylamine, Electrode: GCE: glassy carbon electrode, CPE: carbon paste electrode, PGE: pencil graphite electrode, SPCE: screen-printed carbon electrode, Method: DPV: differential pulse voltammetry, CV: cyclic voltammetry, SWV: square wave voltammetry, PCR: polymerase chain reaction

## References

[1] Wang J (2006). Electrochemical biosensors: towards point-of-care cancer diagnostics. Biosensors and Bioelectronics.

[2] Grieshaber D, MacKenzie R, Vörös J, Reimhult E (2008). Electrochemical biosensors-sensor principles and architectures. Sensors.

[3] Zhang C, Du X (2020). Electrochemical sensors based on carbon nanomaterial used in diagnosing metabolic disease. Frontiers in Chemistry.

[4] Huang X, Zhu Y, Kianfar E (2021). Nano biosensors: properties, applications and electrochemical techniques. Journal of Materials Research and Technology.

[5] Aflatoonian MR, Tajik S, Aflatoonian B, Ekrami-Kakhki MS, Divsalar K (2020). Development of a new electrochemical sensor based on modified carbon paste electrode for simultaneous determination of norepinephrine and acetaminophen in real samples. Eurasian Chemical Communications.

[6] Chupradit S, Nasution MK, Rahman HS, Suksatan W, Jalil AT (2022). Various types of electrochemical biosensors for leukemia detection and therapeutic approaches. Analytical Biochemistry.

[7] Wang J (2023). Analytical Electrochemistry,.

[8] Luppa PB, Sokoll LJ, Chan DW (2001). Immunosensors—principles and applications to clinical chemistry. Clinica Chimica Acta.

[9] Wang J (2002). Electrochemical nucleic acid biosensors. Analytica Chimica Acta.

[10] Erdem A, Ozsoz M (2002). Electrochemical DNA biosensors based on DNA-drug interactions. Electroanalysis: An International Journal Devoted to Fundamental and Practical Aspects of Electroanalysis.

[11] Paleček E (1960). Oscillographic polarography of highly polymerized deoxyribonucleic acid. Nature.

[12] Erdem A, Özsöz M (2001). Voltammetry of the anticancer drug mitoxantrone and DNA. Turkish Journal of Chemistry.

[13] Erdem A, Kosmider B, Osiecka R, Zyner E, Ochocki J (2005). Electrochemical genosensing of the interaction between the potential chemotherapeutic agent, *cis*-bis(3-aminoflavone)dichloroplatinum(II) and DNA in comparison with *cis*-DDP. Journal of Pharmaceutical and Biomedical Analysis.

[14] Canavar PE, Ekşin E, Gürsan KAE (2015). Electrochemical monitoring of the interaction between mitomycin C and DNA at chitosan–carbon nanotube composite modified electrodes. Turkish Journal of Chemistry.

[15] Jelen F, Erdem A, Paleček E (2002). Cyclic voltammetry of echinomycin and its interaction with double-stranded and single-stranded DNA adsorbed at the electrode. Bioelectrochemistry.

[16] Ilkhani H, Hughes T, Li J, Zhong CJ, Hepel M (2016). Nanostructured SERS-electrochemical biosensors for testing of anticancer drug interactions with DNA. Biosensors and Bioelectronics.

[17] Sadat Vajedi F, Dehghani H (2020). A high-sensitive electrochemical DNA biosensor based on a novel ZnAl/layered double hydroxide modified cobalt ferrite-graphene oxide nanocomposite electrophoretically deposited onto FTO substrate for electroanalytical studies of etoposide. Talanta.

[18] Kurbanoglu S, Dogan-Topal B, Hlavata L, Labuda J, Ozkan SA (2015). Electrochemical investigation of an interaction of the antidepressant drug aripiprazole with original and damaged calf thymus dsDNA. Electrochimica Acta.

[19] Vaníčková M, Lehotay J, Čižmárik J, Labuda J (2005). Kinetic study of the degradation of a potential local anesthetic drug in serum using the DNA-based electrochemical biosensor. Bioelectrochemistry.

[20] Radi AE, El-Naggar AE, Nassef HM (2014). Electrochemical and spectral studies on the interaction of the antiparasitic drug nitazoxanide with DNA. Electrochimica Acta.

[21] Morawska K, Popławski T, Ciesielski W, Smarzewska S (2018). Electrochemical and spectroscopic studies of the interaction of antiviral drug Tenofovir with single and double stranded DNA. Bioelectrochemistry.

[22] Machini WBS, Fernandes IPG, Oliveira Brett AM (2019). Antidiabetic drug metformin oxidation and *in situ* interaction with dsDNA using a dsDNA-electrochemical Biosensor. Electroanalysis.

[23] Nemčeková K, Labuda J, Milata V, Blaškovičová J, Sochr J (2018). Interaction of DNA and mononucleotides with theophylline investigated using electrochemical biosensors and biosensing. Bioelectrochemistry.

[24] Erdem A, Ozsoz M (2001). Interaction of the anticancer drug epirubicin with DNA. Analytica Chimica Acta.

[25] Erdem A, Karadeniz H, Caliskan A (2009). Single-walled carbon nanotubes modified graphite electrodes for electrochemical monitoring of nucleic acids and biomolecular interactions. Electroanalysis: An International Journal Devoted to Fundamental and Practical Aspects of Electroanalysis.

[26] Erdem A, Congur G (2013). Impedimetric detection of in situ interaction between anti-cancer drug bleomycin and DNA. International Journal of Biological Macromolecules.

[27] Yapasan E, Caliskan A, Karadeniz H, Erdem A (2010). Electrochemical investigation of biomolecular interactions between platinum derivatives and DNA by carbon nanotubes modified sensors. Materials Science and Engineering: B.

[28] De la Cruz Morales K, Alarcón-Angeles G, Merkoçi A (2019). Nanomaterial-based sensors for the study of DNA interaction with drugs. Electroanalysis.

[29] Aleksić M, Kapetanović V (2014). An overview of the optical and electrochemical methods for detection of DNA-drug interactions. Acta Chimica Slovenica.

[30] Ramotowska S, Ciesielska A, Makowski M (2021). What can electrochemical methods offer in determining DNA–drug interactions?. Molecules.

[31] Eksin E, Senturk H, Zor E, Bingol H, Erdem A (2020). Carbon quantum dot modified electrodes developed for electrochemical monitoring of Daunorubicin-DNA interaction. Journal of Electroanalytical Chemistry.

[32] Nimal R, Unal DN, Erkmen C, Bozal Palabiyik B, Siddiq M (2022). Development of the electrochemical, spectroscopic and molecular docking approaches toward the investigation of interaction between DNA and anti-leukemic drug azacytidine. Bioelectrochemistry.

[33] Karimi Maleh H, Khataee A, Karimi F, Baghayeri M, Fu L (2022). A green and sensitive guanine-based DNA biosensor for idarubicin anticancer monitoring in biological samples: a simple and fast strategy for control of health quality in chemotherapy procedure confirmed by docking investigation. Chemosphere.

[34] Findik M, Bingol H, Erdem A (2021). Hybrid nanoflowers modified pencil graphite electrodes developed for electrochemical monitoring of interaction between Mitomycin C and DNA. Talanta.

[35] Congur G, Eksin E, Erdem A (2021). Levan modified DNA biosensor for voltammetric detection of daunorubicin-DNA interaction. Sensors and Actuators B: Chemical.

[36] Porfireva AV, Goida AI, Rogov AM, Evtugyn GA (2020). Impedimetric DNA sensor based on poly (proflavine) for determination of anthracycline drugs. Electroanalysis.

[37] Evtugyn G, Porfireva A, Stepanova V, Budnikov H (2015). Electrochemical biosensors based on native DNA and nanosized mediator for the detection of anthracycline preparations. Electroanalysis.

[38] Mascini M, Bagni G, Pietro MLD, Ravera M, Baracco S (2006). Electrochemical biosensor evaluation of the interaction between DNA and metallo-drugs. Biometals.

[39] Radi A, El Ries MA, Kandil S (2003). Electrochemical study of the interaction of levofloxacin with DNA. Analytica Chimica Acta.

[40] Bilge S, Dogan Topal B, Tok TT, Atici EB, Sınağ A (2022). Investigation of the interaction between anticancer drug ibrutinib and double-stranded DNA by electrochemical and molecular docking techniques. Microchemical Journal.

[41] Rafique B, Khalid AM, Akhtar K, Jabbar A (2013). Interaction of anticancer drug methotrexate with DNA analyzed by electrochemical and spectroscopic methods. Biosensors and Bioelectronics.

[42] Eksin E, Polat D, Erdem A (2019). Voltammetric and impedimetric detection of interaction between dacarbazine and nucleic acids. Electroanalysis.

[43] Katzung BG (2017). Basic & Clinical Pharmacology.

[44] Javar HA, Garkani Nejad Z, Dehghannoudeh G, Mahmoudi Moghaddam H (2020). Development of a new electrochemical DNA biosensor based on Eu^3+/−^ doped NiO for determination of amsacrine as an anti-cancer drug: electrochemical, spectroscopic and docking studies. Analytica Chimica Acta.

[45] Abedi R, Raoof JB, Hashkavayi AB, Asghary M (2021). Highly sensitive and label-free electrochemical biosensor based on gold nanostructures for studying the interaction of prostate cancer gene sequence with epirubicin anti-cancer drug. Microchemical Journal.

[46] Findik M, Bingol H, Erdem A (2021). Electrochemical detection of interaction between daunorubicin and DNA by hybrid nanoflowers modified graphite electrodes. Sensors and Actuators B: Chemical.

[47] Sengiz C, Congur G, Eksin E, Erdem A (2015). Multiwalled carbon nanotubes-chitosan modified single-use biosensors for electrochemical monitoring of drug-DNA interactions. Electroanalysis.

[48] Eksin E, Muti M, Erdem A (2013). Chitosan/ionic liquid composite electrode for electrochemical monitoring of the surface-confined interaction between mitomycin C and DNA. Electroanalysis.

[49] Saljooqi A, Shamspur T, Mostafavi A (2019). The MWCNT-Ag-PT GCE electrochemical sensor functionalized with dsDNA for mitoxantrone sensing in biological media. IEEE Sensors Journal.

[50] Machini WBS, Marques NV, Oliveira-Brett AM (2019). *In situ* evaluation of anticancer monoclonal antibody nivolumab-DNA interaction using a DNA-electrochemical biosensor. ChemElectroChem.

[51] Eksin E, Congur G, Mese F, Erdem A (2014). Electrochemical monitoring of surface confined interaction between 6-thioguanine and DNA by using single-use graphite electrode. Journal of Electroanalytical Chemistry.

[52] Congur G, Erdem A, Mese F (2015). Electrochemical investigation of the interaction between topotecan and DNA at disposable graphite electrodes. Bioelectrochemistry.

[53] Porfireva A, Plastinina K, Evtugyn V, Kuzin Y, Evtugyn G (2021). Electrochemical DNA sensor based on poly(Azure a) obtained from the buffer saturated with chloroform. Sensors.

[54] Porfireva A, Evtugyn G (2020). Electrochemical DNA sensor based on the copolymer of proflavine and Azure B for doxorubicin determination. Nanomaterials.

[55] Kulikova TN, Porfireva AV, Shamagsumova RV, Evtugyn GA (2018). Voltammetric sensor with replaceable polyaniline-DNA layer for doxorubicin determination. Electroanalysis.

[56] Shamagsumova R, Porfireva A, Stepanova V, Osin Y, Evtugyn G (2015). Polyaniline–DNA based sensor for the detection of anthracycline drugs. Sensors and Actuators B: Chemical.

[57] Goida A, Kuzin Y, Evtugyn V, Porfireva A, Evtugyn G (2022). Electrochemical sensing of idarubicin–DNA interaction using electropolymerized Azure B and Methylene blue mediation. Chemosensors.

[58] Unal DN, Eksin E, Erdem A (2017). Carbon nanotubes modified graphite electrodes for monitoring of biointeraction between 6-thioguanine and DNA. Electroanalysis.

[59] Song KX, Chen D, Liao ZY, Yu X, Guo FF (2022). Label-free electrochemical detection of genetic damage induced by the interaction of a novel potential aminoanthraquinone-derived antitumor agent with DNA modified electrode. Sensors and Actuators B: Chemical.

[60] Meric B, Kerman K, Ozkan D, Kara P, Erdem A (2002). Electrochemical biosensor for the interaction of DNA with the alkylating agent 4,4′-dihydroxy chalcone based on guanine and adenine signals. Journal of Pharmaceutical and Biomedical Analysis.

[61] Zeybek DK, Demir B, Zeybek B, Pekyardımcı Ş (2015). A sensitive electrochemical DNA biosensor for antineoplastic drug 5-fluorouracil based on glassy carbon electrode modified with poly(bromocresol purple). Talanta.

[62] Piedade JAP, Fernandes IR, Oliveira Brett AM (2002). Electrochemical sensing of DNA–adriamycin interactions. Bioelectrochemistry.

[63] Tomé LI, Marques NV, Diculescu VC, Oliveira-Brett AM (2015). *In situ* dsDNA-bevacizumab anticancer monoclonal antibody interaction electrochemical evaluation. Analytica Chimica Acta.

[64] Heydari-Bafrooei E, Amini M, Saeednia S (2017). Electrochemical detection of DNA damage induced by Bleomycin in the presence of metal ions. Journal of Electroanalytical Chemistry.

[65] Krizkova S, Adam V, Petrlova J, Zitka O, Stejskal K (2007). A suggestion of electrochemical biosensor for study of platinum(II)-DNA interactions. Electroanalysis: An International Journal Devoted to Fundamental and Practical Aspects of Electroanalysis.

[66] Satana HE, Pontinha ADR, Diculescu VC, Oliveira-Brett AM (2012). Nucleoside analogue electrochemical behaviour and in situ evaluation of DNA–clofarabine interaction. Bioelectrochemistry.

[67] Wang S, Wang R, Sellin PJ, Chang S (2009). Carbon nanotube based DNA biosensor for rapid detection of anti-cancer drug of cyclophosphamide. Current Nanoscience.

[68] Foroughi MM, Jahani S, Aramesh-Broujeni Z, Dolatabad MR (2021). A label-free electrochemical biosensor based on 3D cubic Eu^3+^/Cu_2_O nanostructures with clover-like faces for the determination of anticancer drug cytarabine. RSC Advances.

[69] Eksin E, Zor E, Erdem A, Bingol H (2017). Electrochemical monitoring of biointeraction by graphene-based material modified pencil graphite electrode. Biosensors and Bioelectronics.

[70] Congur G, Eksin E, Erdem A (2019). Chitosan modified graphite electrodes developed for electrochemical monitoring of interaction between daunorubicin and DNA. Sensing and Bio-Sensing Research.

[71] Stepanova V, Smolko V, Gorbatchuk V, Stoikov I, Evtugyn G (2019). DNA-polylactide modified biosensor for electrochemical determination of the DNA-drugs and aptamer-aflatoxin M1 interactions. Sensors.

[72] Hajian R, Tayebi Z, Shams N (2017). Fabrication of an electrochemical sensor for determination of doxorubicin in human plasma and its interaction with DNA. Journal of Pharmaceutical Analysis.

[73] Trnkova L, Huska D, Adam V, Kizek R, Eckschlager T (2009). Electrochemical biosensor for investigation of anticancer drugs interactions (doxorubicin and ellipticine) with DNA. IEEE Sensors.

[74] Satana HE, Oliveira Brett AM (2011). *In situ* evaluation of fludarabine-DNA interaction using a DNA-electrochemical biosensor. International Journal of Electrochemistry.

[75] Raoof JB, Bagheryan Z, Hashkavayi AB (2020). Development of a DNA biosensor based on MCM41 modified screen-printed graphite electrode for the study of the short sequence of the p53 tumor suppressor gene in hybridization and its interaction with the flutamide drug using hemin as the electrochemical label. New Journal of Chemistry.

[76] Paimard G, Gholivand MB, Shamsipur M (2016). Determination of ganciclovir as an antiviral drug and its interaction with DNA at Fe_3_O_4_/carboxylated multi-walled carbon nanotubes modified glassy carbon electrode. Measurement.

[77] Shoja Y, Kermanpur A, Karimzadeh F, Ghodsi J, Rafati AA (2019). Electrochemical molecularly bioimprinted siloxane biosensor on the basis of core/shell silver nanoparticles/EGFR exon 21 L858R point mutant gene/siloxane film for ultra-sensing of Gemcitabine as a lung cancer chemotherapy medication. Biosensors and Bioelectronics.

[78] Buoro RM, Lopes IC, Diculescu VC, Serrano SH, Lemos L (2014). *In situ* evaluation of gemcitabine-DNA interaction using a DNA-electrochemical biosensor. Bioelectrochemistry.

[79] Tığ GA, Zeybek B, Pekyardımcı Ş (2016). Electrochemical DNA biosensor based on poly(2,6-pyridinedicarboxylic acid) modified glassy carbon electrode for the determination of anticancer drug gemcitabine. Talanta.

[80] Kara HES (2014). Redox mechanism of anticancer drug idarubicin and in-situ evaluation of interaction with DNA using an electrochemical biosensor. Bioelectrochemistry.

[81] Öndeş B, Muti M (2020). Electrochemical determination of the effect of caffeic acid onto the interaction between idarubicin and DNA by single-use disposable electrodes. Electroanalysis.

[82] Foroughi MM, Jahani S (2022). Investigation of a high-sensitive electrochemical DNA biosensor for determination of Idarubicin and studies of DNA-binding properties. Microchemical Journal.

[83] Bolat G (2020). Investigation of poly(CTAB-MWCNTs) composite based electrochemical DNA biosensor and interaction study with anticancer drug Irinotecan. Microchemical Journal.

[84] Dogan Topal B, Bozal Palabiyik B, Ozkan SA, Uslu B (2014). Investigation of anticancer drug lapatinib and its interaction with dsDNA by electrochemical and spectroscopic techniques. Sensors and Actuators B: Chemical.

[85] Dogan Topal B, Ozkan SA (2011). A novel sensitive electrochemical DNA biosensor for assaying of anticancer drug leuprolide and its adsorptive stripping voltammetric determination. Talanta.

[86] Pontinha ADR, Jorge SMA, Paquim AMC, Diculescu VC, Oliveira-Brett AM (2011). *In situ* evaluation of anticancer drug methotrexate–DNA interaction using a DNA-electrochemical biosensor and AFM characterization. Physical Chemistry Chemical Physic.

[87] Wang F, Wu Y, Liu J, Ye B (2009). DNA Langmuir–Blodgett modified glassy carbon electrode as voltammetric sensor for determinate of methotrexate. Electrochimica Acta.

[88] Karadeniz H, Alparslan L, Erdem A, Karasulu E (2007). Electrochemical investigation of interaction between mitomycin C and DNA in a novel drug-delivery system. Journal of Pharmaceutical and Biomedical Analysis.

[89] Canavar E, Kuralay F, Erdem A (2011). Interaction of mitomycin C with DNA immobilized onto single-walled carbon nanotube/polymer modified pencil graphite electrode. Electroanalysis.

[90] Lad AN, Agrawal YK (2013). Multi-wall carbon nanotube-based DNA nanosensor for determining mitoxantrone-DNA interaction *in-vitro*. Instrumentation Science & Technology.

[91] Chen D, Yu X, Qin Y, Liao ZY, Li T (2022). Electrochemical detection of DNA damage caused by novel potential 2-nitroimidazole naphthalimide-based hypoxia tumor-targeting agent with minimum side effects. Microchemical Journal.

[92] Moarefdoust MM, Jahani S, Moradalizadeh M, Motaghi MM, Foroughi MM (2022). A DNA biosensor based on a raspberry-like hierarchical nano-structure for the determination of the anticancer drug nilotinib. ChemistryOpen.

[93] Mehdinia A, Kazemi SH, Bathaie SZ, Alizadeh A, Shamsipur M (2008). Electrochemical studies of DNA immobilization onto the azide-terminated monolayers and its interaction with taxol. Analytical Biochemistry.

[94] Taei M, Hassanpour F, Salavati H, Sadeghi Z, Alvandi H (2015). Highly selective electrochemical determination of taxol based on ds-DNA-modified pencil electrode. Applied Biochemistry and Biotechnology.

[95] Jahandari S, Taher MA, Karimi-Maleh H, Khodadadi A, Faghih-Mirzaei E (2019). A powerful DNA-based voltammetric biosensor modified with Au nanoparticles, for the determination of Temodal; an electrochemical and docking investigation. Journal of Electroanalytical Chemistry.

[96] Topkaya SN, Serindere G, Ozder M (2016). Determination of DNA hypermethylation using anti-cancer drug-Temozolomide. Electroanalysis.

[97] Top M, Er O, Congur G, Erdem A, Lambrecht FY (2016). Intracellular uptake study of radiolabeled anticancer drug and impedimetric detection of its interaction with DNA. Talanta.

[98] Beitollahi H, Dehghannoudeh G, Moghaddam HM, Forootanfar H (2017). A sensitive electrochemical DNA biosensor for anticancer drug topotecan based on graphene carbon paste electrode. Journal of The Electrochemical Society.

[99] Hajian R, Mehrayin Z, Mohagheghian M, Zafari M, Hosseini P (2015). Fabrication of an electrochemical sensor based on carbon nanotubes modified with gold nanoparticles for determination of valrubicin as a chemotherapy drug: Valrubicin-DNA interaction. Materials Science and Engineering: C.

[100] Al Jawadi EAM, Majeed MI (2021). Detection of anticancer drug by electrochemical sensors at modified electrode (MWCNT/polyEosin-Y). Nanomedicine Research Journal.

[101] Ensafi AA, Heydari Bafrooei E, Rezaei B (2013). Different interaction of codeine and morphine with DNA: a concept for simultaneous determination. Biosensors and Bioelectronics.

[102] Asghary M, Raoof JB, Ojani R, Hamidi Asl E (2015). A genosensor based on CPE for study the interaction between ketamine as an anesthesia drug with DNA. International Journal of Biological Macromolecules.

[103] Nimal R, Unal DN, Erkmen C, Kurbanoglu S, Siddiq M (2023). Elucidating the interaction of antidepressant drug paroxetine with ct-dsDNA: a comparative study by electrochemical, spectroscopic, and molecular docking approaches. Bioelectrochemistry.

[104] Finberg RW, Moellering RC, Tally FP, Craig WA, Pankey GA (2004). The importance of bactericidal drugs: future directions in infectious disease. Clinical Infectious Diseases.

[105] Nemeth J, Oesch G, Kuster SP (2015). Bacteriostatic versus bactericidal antibiotics for patients with serious bacterial infections: systematic review and meta-analysis. Journal of Antimicrobial Chemotherapy.

[106] Topkaya SN, Cetin AE (2019). Determination of electrochemical interaction between 2-(1H-benzimidazol-2-yl) phenol and DNA sequences. Electroanalysis.

[107] Nawaz H, Rauf S, Akhtar K, Khalid AM (2006). Electrochemical DNA biosensor for the study of ciprofloxacin–DNA interaction. Analytical Biochemistry.

[108] Cheraghi S, Taher MA, Karimi Maleh H, Faghih Mirzaei E (2017). A nanostructure label-free DNA biosensor for ciprofloxacin analysis as a chemotherapeutic agent: an experimental and theoretical investigation. New Journal of Chemistry.

[109] Diab N, Abu Shqair I, Salim R, Al Subu M (2014). The behavior of ciprofloxacin at a DNA modified glassy carbon electrodes. International Journal of Electrochemical Science.

[110] Mahmoudi-Moghaddam H, Garkani-Nejad Z (2022). Development of the electrochemical biosensor for determination of antibiotic drug isoniazid using ds-DNA/Carbon/La^3+^/CuO/CPE. Measurement.

[111] Aydoğdu G, Günendi G, Zeybek DK, Zeybek B, Pekyardımcı Ş (2014). A novel electrochemical DNA biosensor based on poly-(5-amino-2-mercapto-1, 3, 4-thiadiazole) modified glassy carbon electrode for the determination of nitrofurantoin. Sensors and Actuators B: Chemical.

[112] Li CZ, Liu Y, Luong JH (2005). Impedance sensing of DNA binding drugs using gold substrates modified with gold nanoparticles. Analytical Chemistry.

[113] Shumyantseva VV, Bulko TV, Tikhonova EG, Sanzhakov MA, Kuzikov AV (2021). Electrochemical studies of the interaction of rifampicin and nanosome/rifampicin with dsDNA. Bioelectrochemistry.

[114] Qian L, Durairaj S, Prins S, Chen A (2021). Nanomaterial-based electrochemical sensors and biosensors for the detection of pharmaceutical compounds. Biosensors and Bioelectronics.

[115] Karimi Maleh H, Bananezhad A, Ganjali MR, Norouzi P, Sadrnia A (2018). Surface amplification of pencil graphite electrode with polypyrrole and reduced graphene oxide for fabrication of a guanine/adenine DNA based electrochemical biosensors for determination of didanosine anticancer drug. Applied Surface Science.

[116] Karadeniz H, Gulmez B, Sahinci F, Erdem A, Kaya GI (2003). Disposable electrochemical biosensor for the detection of the interaction between DNA and lycorine based on guanine and adenine signals. Journal of Pharmaceutical and Biomedical Analysis.

[117] Di Santo N, Ehrisman J (2013). Research perspective: potential role of nitazoxanide in ovarian cancer treatment. Old drug, new purpose?. Cancers.

[118] Mehmandoust M, Soylak M, Erk N (2023). Innovative molecularly imprinted electrochemical sensor for the nanomolar detection of Tenofovir as an anti-HIV drug. Talanta.

[119] La Scalea MA, Serrano SHP, Ferreira EI, Brett AO (2002). Voltammetric behavior of benznidazole at a DNA-electrochemical biosensor. Journal of Pharmaceutical and Biomedical Analysis.

[120] Yilmaz N, Eksin E, Karacicek B, Eraç Y, Erdem A (2017). Electrochemical detection of interaction between capsaicin and nucleic acids in comparison to agarose gel electrophoresis. Analytical Biochemistry.

[121] Ensafi AA, Sohrabi M, Jafari-Asl M, Rezaei B (2015). Selective and sensitive furazolidone biosensor based on DNA-modified TiO_2_-reduced graphene oxide. Applied Surface Science.

[122] Shahzad S, Dogan Topal B, Karadurmus L, Caglayan MG, Tok TT (2019). Electrochemical, spectroscopic and molecular docking studies on the interaction of calcium channel blockers with dsDNA. Bioelectrochemistry.

[123] Radi AE, Nassef HM, Attallah MI (2015). Investigation of antimalarial drug pyrimethamine and its interaction with dsDNA by electrochemical and spectroscopic techniques. Analytical Methods.

[124] Gu T, Hasebe Y (2012). Novel amperometric assay for drug–DNA interaction based on an inhibitory effect on an electrocatalytic activity of DNA–Cu (II) complex. Biosensors and Bioelectronics.

[125] Cinková K, Zbojeková N, Vojs M, Marton M, Samphao A (2015). Electroanalytical application of a boron-doped diamond electrode for sensitive voltammetric determination of theophylline in pharmaceutical dosages and human urine. Analytical Methods.

[126] Tocher JH (1997). Reductive activation of nitroheterocyclic compounds. General Pharmacology.

